# Evolutionary Novelty in a Butterfly Wing Pattern through Enhancer Shuffling

**DOI:** 10.1371/journal.pbio.1002353

**Published:** 2016-01-15

**Authors:** Richard W. R. Wallbank, Simon W. Baxter, Carolina Pardo-Diaz, Joseph J. Hanly, Simon H. Martin, James Mallet, Kanchon K. Dasmahapatra, Camilo Salazar, Mathieu Joron, Nicola Nadeau, W. Owen McMillan, Chris D. Jiggins

**Affiliations:** 1 Department of Zoology, University of Cambridge, Cambridge, United Kingdom; 2 Smithsonian Tropical Research Institution, Balboa, Ancón, Panama; 3 School of Biological Sciences, University of Adelaide, Adelaide, Australia; 4 Biology Program, Faculty of Natural Sciences and Mathematics, Universidad del Rosario, Bogotá, D.C., Colombia; 5 Organismic and Evolutionary Biology, Harvard University, Harvard, Massachusetts, United States of America; 6 Department of Biology, University of York, York, United Kingdom; 7 Institut de Systématique Evolution et Biodiversité, UMR 7205, CNRS MNHN UPMC EPHE, Muséum National d'Histoire Naturelle, CP50, Paris, France; 8 Centre d’Ecologie Fonctionnelle et Evolutive, UMR 5175, CNRS–Université de Montpellier–Université Paul-Valéry–EPHE, Montpellier, France; 9 Dept. of Animal and Plant Sciences, University of Sheffield, Sheffield, United Kingdom; Institute of Science and Technology Austria (IST Austria), AUSTRIA

## Abstract

An important goal in evolutionary biology is to understand the genetic changes underlying novel morphological structures. We investigated the origins of a complex wing pattern found among Amazonian *Heliconius* butterflies. Genome sequence data from 142 individuals across 17 species identified narrow regions associated with two distinct red colour pattern elements, *dennis* and *ray*. We hypothesise that these modules in non-coding sequence represent distinct *cis*-regulatory loci that control expression of the transcription factor *optix*, which in turn controls red pattern variation across *Heliconius*. Phylogenetic analysis of the two elements demonstrated that they have distinct evolutionary histories and that novel adaptive morphological variation was created by shuffling these *cis*-regulatory modules through recombination between divergent lineages. In addition, recombination of modules into different combinations within species further contributes to diversity. Analysis of the timing of diversification in these two regions supports the hypothesis of introgression moving regulatory modules between species, rather than shared ancestral variation. The dennis phenotype introgressed into *Heliconius melpomene* at about the same time that ray originated in this group, while ray introgressed back into *H*. *elevatus* much more recently. We show that shuffling of existing enhancer elements both within and between species provides a mechanism for rapid diversification and generation of novel morphological combinations during adaptive radiation.

## Abstract 2

Novel butterfly wing patterns have arisen via the sharing of gene regulatory elements between lineages through introgression and recombination; new combinations of regulatory elements offer a rapid route to the evolution of new patterns.

## Author Summary

Butterflies show an amazing diversity of patterns on their wings. In fact, most of the 18,000 species of butterfly can be distinguished on the basis of their wing pattern. Much of this diversity is thought to arise through novel switches in the genome that turn genes on in new contexts during wing development, thereby producing new patterns. Here we study a set of switches that control the expression of *optix*, a gene that places red patches onto the wings of *Heliconius* butterflies. We show that two patterning switches—one that produces red rays on the hindwing and the other a red patch on the base of the forewing—are located adjacent to one another in the genome. These switches have each evolved just once among a group of 16 species but have then been repeatedly shared between species by hybridisation and introgression. Despite the fact that they are now part of a common pattern in the Amazon basin, these two pattern components actually arose in completely different species before being brought together through hybridisation. In addition, recombination among these switches has produced new combinations of patterns within species. Such sharing of genetic variation is one way in which mimicry can evolve, whereby patterns are shared between species to send a common signal to predators. Our work suggests a new mechanism for generating evolutionary novelty, by shuffling these genetic switches among lineages and within species.

## Introduction

One of the major impediments to evolutionary innovation is the constraint on genetic change imposed by existing function [1]. Mutations that confer advantageous phenotypic effects in a novel trait will often result in negative pleiotropic effects in other traits influenced by the same gene. Several mechanisms have been proposed by which evolution can circumvent such constraints, resulting in phenotypic diversification. In particular, the modularity of *cis*-regulatory elements [2–6] means that novel modules can encode new expression domains and functions without disrupting existing expression patterns [6,7]. This modularity underlying gene regulation has led to the assertion that much of morphological diversity has arisen through regulatory evolution [6].

Much of our understanding of modularity in regulatory evolution comes from *Drosophila*, in which the loss of trichomes on the larval cuticle [5], the gain of melanic wing spots [8–10], or changes in abdominal pigmentation [3,11] have been shown to involve evolutionary changes in *cis*-regulatory elements. These elegant developmental studies demonstrate the underlying logic of regulatory modularity, whereby novel expression domains can arise without disrupting existing function. These studies have also established a paradigm in which small effect mutations alter transcription factor binding sites in these regulatory modules and in combination produce large effect alleles [5]. Similar conclusions come from recent work in other taxa, including mice and jewel wasps [2,12]. This might seem to imply that the evolution of novel regulatory alleles is relatively gradual, requiring the evolution of many small effect substitutions, but recent adaptive radiations can show extremely rapid rates of morphological change. The role of regulatory modularity therefore remains to be tested in adaptive radiations in which morphological variation evolves very rapidly.

**Fig 1:**
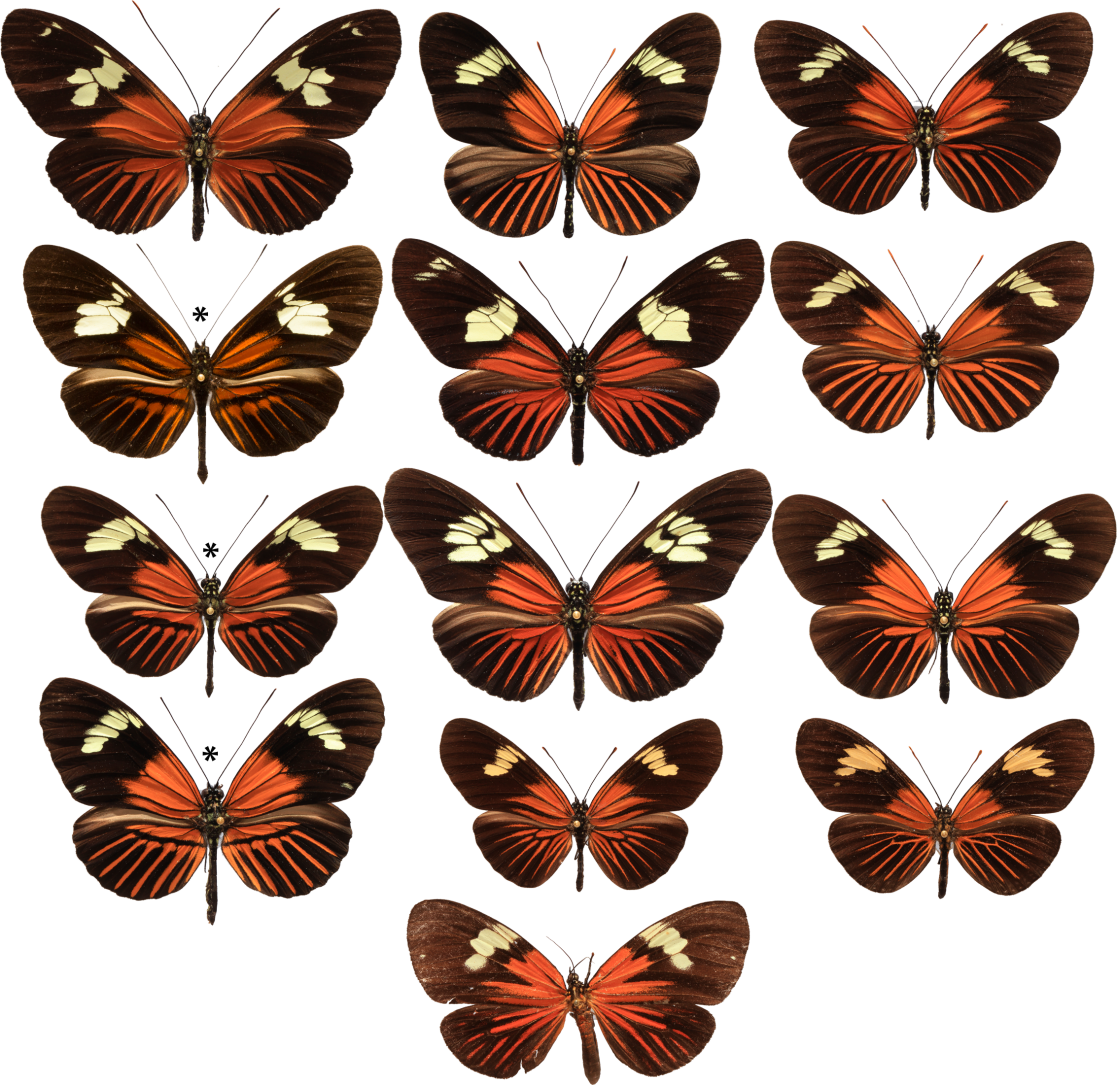
Diversity of the Amazonian dennis-ray mimicry ring. First row: *H*. *burneyi huebneri*, *H*. *aoede auca*, and *H*. *xanthocles zamora;* second row: *H*. *timareta timareta* f. *timareta*, *H*. *doris doris*, and *H*. *demeter ucayalensis;* third row: *H*. *melpomene malleti*, *H*. *egeria homogena*, and *H*. *erato emma*; fourth row: *H*. *elevatus pseudocupidineus*, *Eueides heliconioides eanes*, and *E*. *tales calathus*; and bottom: *Chetone phyleis*, a pericopine moth. Stars indicate the three species that are the focus of this study. Butterflies figured are from the Neukirchen Collection, McGuire Centre, Florida. The butterflies are from populations in both Ecuador and Peru.

Here we explore the origins of adaptive novelty among the wing patterns of *Heliconius* butterflies. These wing patterns are under strong natural selection for mimicry and warning colour, as well as being important mating signals [13]. The rapid radiation in *Heliconius* is accompanied by an even more rapid diversification in mimicry patterns as well as convergence among species found in a given locality [14], both through independent convergent evolution and via introgression of gene regions between races and species [15,16]. Mimetic convergence reaches its peak among red dennis-ray pattern phenotypes in the Amazon (Fig 1), where 11 or more *Heliconius* species, as well as pierine butterflies and pericopine moths, share the same pattern. In addition to near perfect convergence in wing patterns in a given locality, there is also often striking divergence of patterns between localities as populations adapt to the many different mimicry complexes spread across the Neotropics [17]. This diversity provides an opportunity to study the genetic and developmental basis of evolutionary novelty.

**Fig 2:**
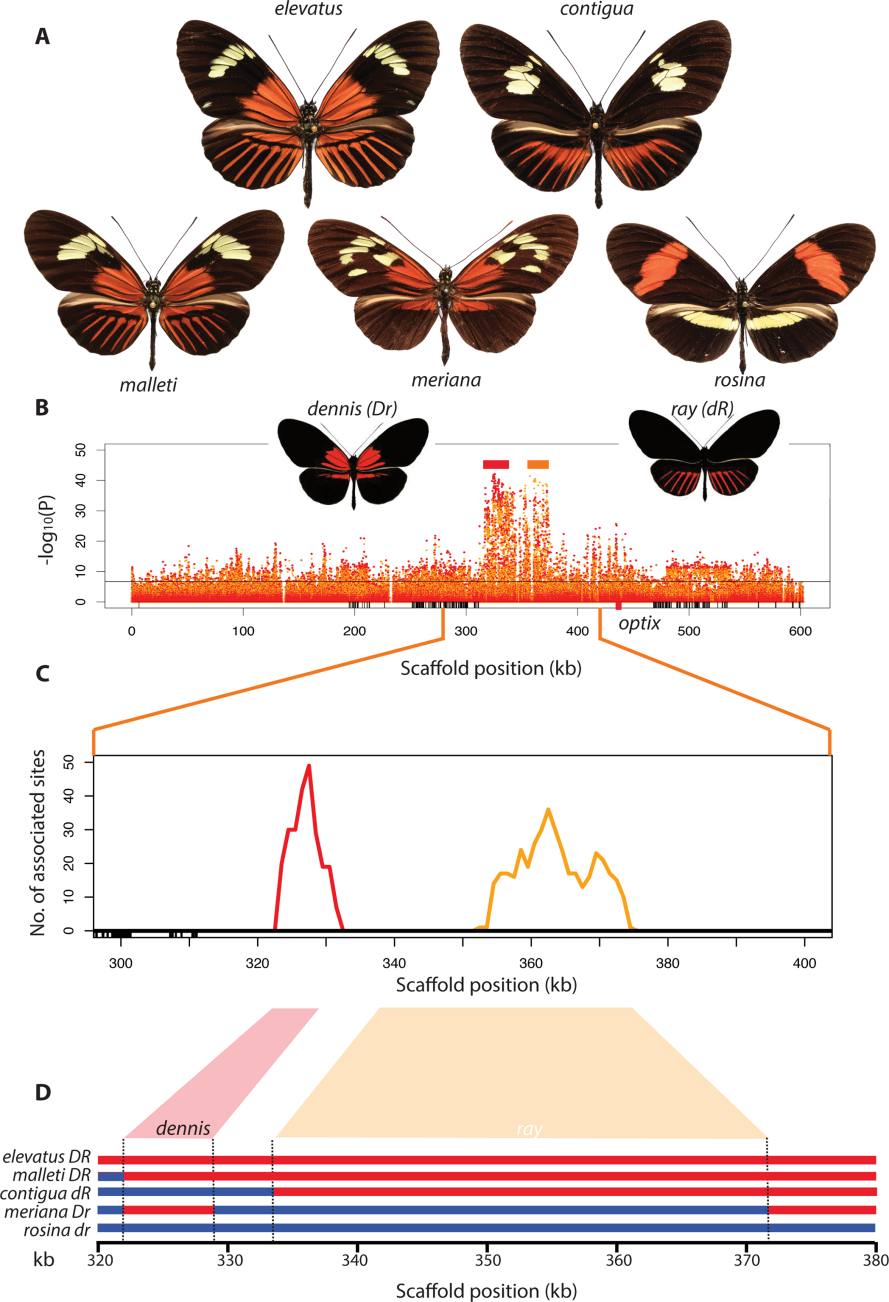
Genotype-by-phenotype association analyses and mapping of *dennis* and *ray* regions. (A) Examples of the principal red wing patterns: dennis-ray *H*. *e*. *pseudocupidineus* and *H*. *m*. *malleti*, dennis-only *H*. *m*. *meriana*, ray-only *H*. *t*. *timareta* f. *contigua*, and band *H*. *m*. *rosina*. (B) Association analysis across 96 genomes showing statistical association for the dennis (red dots) and ray phenotypes (orange dots). The horizontal black line represents significance after Bonferroni correction. Boxes show exon positions. (C) Sliding window analysis of fixed differences between specific comparisons to identify dennis- and ray-associated sites (orange and red lines—see also S1 Fig and S1 Table). (D) Recombination breakpoints allowed separate isolation of regions fully associated with dennis and ray phenotypes. Informative haplotypes are shown (*H*. *elevatus*, *H*. *m*. *malleti*, *H*. *t*. *timareta* f. *contigua*, *H*. *m*. *meriana*, and *H*. *m*. *rosina*, phenotypes shown above). Genotypes are indicated as D/d for *dennis* present/absent and R/r for *ray* present/absent. A *H*. *melpomene* recombinant hybrid was heterozygous in the *dennis* region and homozygous for *ray*-absent, as expected, but was not informative for precise breakpoint delineation because of missing data. See Dryad depository for plot data [32].

Generally, the patterns on butterfly wings are a good system in which to link genetic changes to the developmental processes that generate diversity [18,19]. Wing colour patterns are mosaics of scales, each with a single colour, produced by a combination of pigment and ultrastructure. The relative positions of differently coloured scales are established during larval and pupal wing development [20]. Wing development is thought to be broadly conserved in insects, with wing developmental genes showing similar expression patterns between flies and butterflies [21–23]. This therefore raises the question: how is this conserved landscape of wing development translated into the diversity of butterfly wing patterns? In *Heliconius*, pattern diversity is controlled by a surprisingly small number of genomic regions with large effect sizes [24,25]. In particular, genetic mapping and gene expression studies have shown that red elements are associated with expression of the transcription factor *optix* across all *Heliconius* species [26,27]. In the absence of fixed coding sequence changes between wing pattern forms, this implies that red pattern variation is controlled by differential regulatory control of *optix* [27]. Population genomic studies have identified a region of non-coding sequence downstream of *optix* that is associated with phenotypic change [15,28]. Previous work suggests that there may be several distinct elements within this region. Occasional hybrid phenotypes possess only the “dennis” patch on the base of the forewing or the “ray” elements on the hindwing and have been hypothesised to be rare recombinants, although this has never been tested genetically [29,30]. Similarly, there are also established forms that exhibit only dennis or ray patterns (*H*. *melpomene meriana* and *H*. *timareta timareta* f. *contigua*, respectively, Fig 2). This suggests that the broad genomic interval already identified might contain discrete regulatory loci that vary the spatial expression of *optix* in different wing regions, a hypothesis that we can now test with genetic data.

Here we focus on the *H*. *melpomene* lineage, in which the Amazonian dennis-ray phenotype has evolved recently from a red-banded ancestor [31]. We carry out a population genomic analysis on *H*. *melpomene* and its relatives, *H*. *elevatus* and *H*. *timareta*, to identify putative regulatory modules associated with distinct red pattern elements. Previously, population genetic evidence has suggested that mimicry among *H*. *melpomene*, *H*. *elevatus*, and *H*. *timareta* has evolved through sharing of the *dennis-ray* allele by repeated adaptive introgression at the *optix* locus [16]. This is especially surprising in the case of *H*. *elevatus*, which forms part of the “silvaniform” clade that diverged from *H*. *melpomene* around 4 million years ago [14]. Our analysis here indicates that the origin of the red pattern elements is considerably more complex than has been previously supposed, with the dennis and ray elements of the widespread dennis-ray pattern having distinct evolutionary origins in different clades within the genus.

## Results and Discussion

We took advantage of natural phenotypic variants in which the two red elements, dennis and ray, occur separately to identify putative functional regulatory regions controlling red pattern within the *H*. *melpomene* clade. Genomic analysis of 96 individuals from the *melpomene-timareta* clade revealed two distinct regions that showed strong association with the dennis and ray pattern elements, respectively. Our analysis included a race of *H*. *melpomene*, *H*. *m*. *meriana*, from the Guiana shield, which possesses the forewing dennis patch but not ray, as well as *H*. *t*. *timareta* f. *contigua* from Ecuador, which possesses ray but not dennis, plus a recombinant individual from an *H*. *melpomene* hybrid zone in Ecuador with dennis but not ray (Fig 2A). Across all 96 individuals, there were significant genotype-by-phenotype associations across all genome regions surveyed. This “background” signal of genotype-by-phenotype association is likely due to the presence of genetically divergent species in our dataset that are to some degree confounded with phenotype. Nonetheless, our analysis identified a peak of genotype-by-phenotype association spanning roughly 50 kb and located from 60–110 kb 3ʹ of the *optix* gene, similar to what has been observed previously (Fig 2B) [28]. This region also corresponds closely to that identified recently in the mimetic species *H*. *erato* [15], implying convergence in the regulatory architecture controlling wing pattern mimicry at a finer scale than has been previously demonstrated [28,31].

Furthermore, within this region in our data, distinct adjacent peaks of association were observed for the dennis and ray elements. Focusing specifically on fixed single nucleotide polymorphism (SNP) differences between alternative red phenotypes revealed two distinct peaks of association (Fig 2C). One, approximately 10 kb in length, contained SNPs perfectly associated with the red dennis patch. The other adjacent region was broader, roughly 25 kb, and contained SNPs perfectly associated with red hindwing rays.

**Fig 3:**
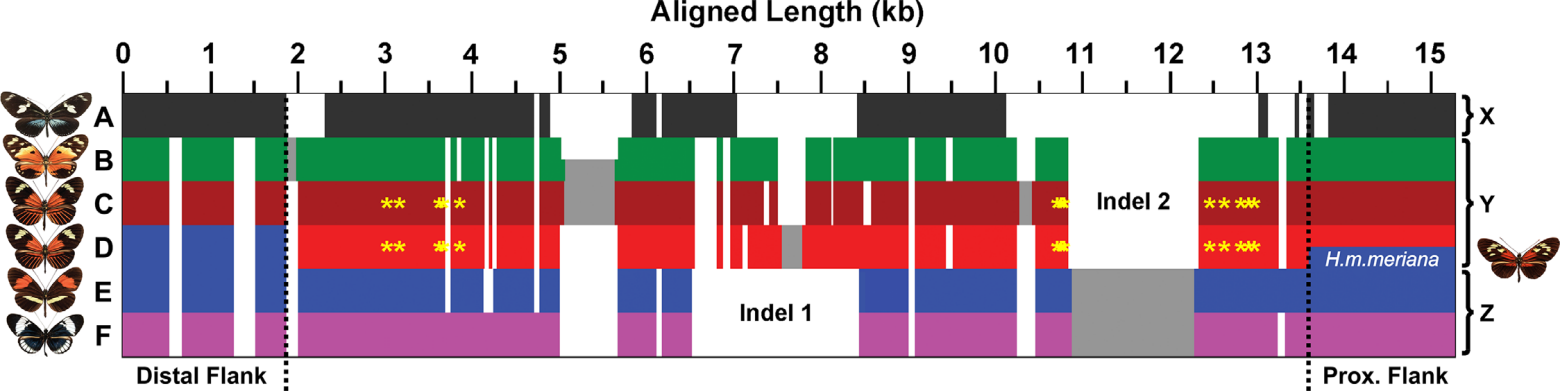
Schematic overview of the *dennis* region alignment architecture. Each group of alleles is characterised by a complex structure of insertion and deletion variation. Horizontal bars represent aligned allele sequences for the outgroups (A), silvaniforms (B), *H*. *elevatus* (C), dennis morph melpomene/timareta (D), non-dennis morph melpomene/timareta (E), and the cydno/heurippa/pachinus clade (F). There are three general forms of the region: an “outgroup” allele (X) with many indels and lacking large sections of sequence; a silvaniform/dennis allele (Y), which possesses indel 1 and lacks indel 2; and a non-dennis/cydno allele (Z), which lacks indel 1 and possesses indel 2. Dotted lines and blue colouration indicate the point where dennis-morph melpomene/timareta alleles become silvaniform like (left) and where the dennis-only morph, *H*. *m*. *meriana*, recombines with the other non-rayed melpomene (right). Fixed SNPs in perfect association with the dennis phenotype are indicated by yellow asterisks and form two clusters. See Dryad depository for alignment [32].

We next used broader taxonomic sampling to further refine these intervals and identify exact sequence haplotypes associated with each of the two phenotypic elements (Fig 2D). To identify recombination breakpoints around *dennis* and *ray* haplotypes, we generated a high-quality sequence alignment by de novo assembly of each individual genome and then identified contigs across the associated region using the Basic Local Alignment Search Tool (BLAST). For the *dennis* region, alignment was assisted by a sequenced fosmid clone from *H*. *m*. *aglaope* (dennis phenotype) to complement the reference genome (derived from a non-dennis butterfly). The final alignment included the 96 *melpomene-timareta* individuals used for association analysis and a further 46 individuals that included species with no red (*H*. *cydno*) and species from the more distantly related silvaniform clade including *H*. *elevatus*, which has the dennis-ray pattern. The distal end of the *dennis* region, relative to *optix*, was delineated by a rapid loss of phenotype-associated variants across all species sampled, whilst the proximal end was determined by a single fixed recombination event in the race *H*. *m*. *meriana* (dennis but no ray phenotype), generating a region of ~7 kb fully associated with dennis. For *ray*, a breakpoint in the ray-only *H*. *t*. *timareta* f. *contigua* defined the distal end, whilst a recombination in *H*. *m*. *meriana* defined the proximal end, resulting in a larger ~37 kb region (Fig 2D). Each haplotype group was characterised by diagnostic SNPs as well as a fixed architecture of indel variation (Fig 3). These analyses therefore support the hypothesis derived from phenotypic evidence, that dennis and ray phenotypes are controlled by adjacent distinct genetic elements. In combination with previous work showing differential expression of *optix* across a wide diversity of *Heliconius* species and races, this provides clear genetic evidence for modularity in the *cis-*regulatory control of *optix*.

**Fig 4:**
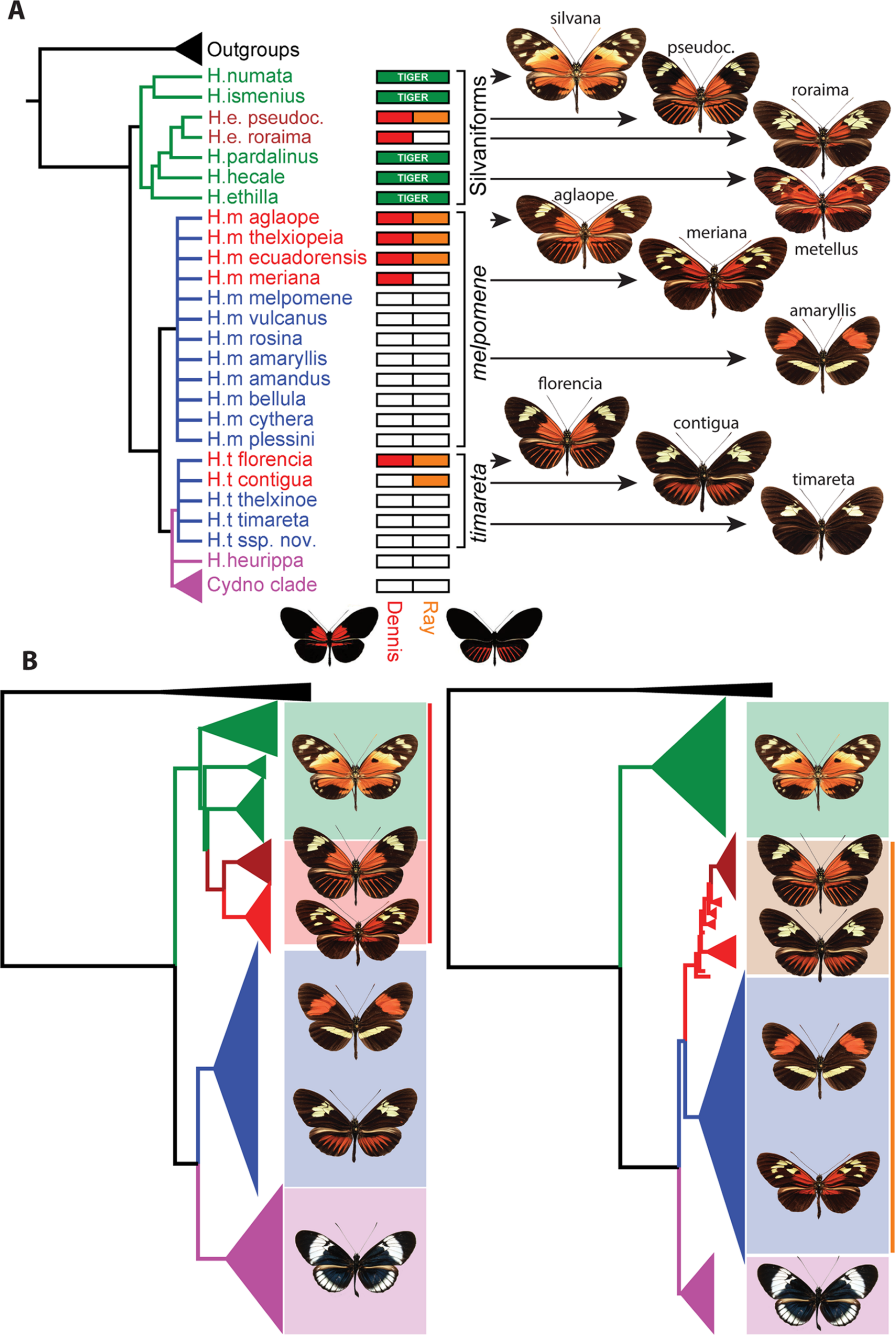
Modularity of *dennis* and *ray* and phylogenetic relationships among alleles at both loci. (A) The phylogeny of species used in this study (left, from [14]) and their respective combinations of *dennis* and *ray* haplotypes (right). Note that although genetic data for *Heliconius elevatus roraima* are not analysed here or included in our alignment, this taxon is shown for completeness. Representative butterfly phenotypes are shown with their respective subspecies or form names. *H*. *e*. *pseudocupidineus* is abbreviated to pseudoc. (B) ML trees with bootstraps (generated using de novo assembled genomes) show that the *dennis* alleles cluster with the silvaniform clade (left), whilst the *ray* alleles cluster with the melpomene/timareta clade (right). Terminal nodes are coloured by silvaniform clade (green, except *H*. *elevatus*), dennis/ray (red, *H*. *melpomene/timareta*; burgundy, *H*. *elevatus*), *melpomene/timareta* non-dennis/ray forms (blue), and *cydno/pachinus/heurippa* clade (purple). Outgroup species are in black (see also S2 Fig for sample labels). *H*. *m*. *meriana* and *H*. *t*. *timareta* f. *contigua* have dennis-only and ray-only patterns, respectively, and cluster with their expected phenotypes. All trees were rooted to *H*. *aoede*. Bootstraps are given as percentages of 1,000 iterations.

We have previously hypothesised that the *dennis-ray* mimicry pattern introgressed as a single genomic block between *H*. *melpomene* and *H*. *timareta*, as well as more distantly between *H*. *melpomene* and *H*. *elevatus* [16]. Our new data suggest a much more complex history than previously recognised, with *dennis* and *ray* having quite distinct origins. As expected, a maximum likelihood (ML) phylogeny shows that the *ray* alleles fall within the *H*. *melpomene* clade, indicating an origin derived from an ancestral *H*. *melpomene* phenotype. In contrast, however, alleles producing the dennis phenotype originated within the silvaniform clade, which diverged from *H*. *melpomene* around 4 million years ago (Fig 4) [14]. Members of this clade have mottled orange/red, black, and yellow “tiger” patterns and are mostly co-mimics of butterflies in the tribe Ithomiini, whereas the melpomene-cydno clade are all co-mimics of other *Heliconius* species. Nonetheless, the silvaniforms commonly have orange patches on the base of the forewing, which in some cases are remarkably similar to the dennis patch of *H*. *melpomene*. In particular, the form *H*. *hecale metellus* has a *dennis*-like phenotype (Fig 4A), which suggests a plausible ancestral phenotype that might have provided the source of the *dennis* allele in *H*. *melpomene*.

Various scenarios might explain this complex history. Sharing of variation between species can be explained by either retention of ancestral polymorphism or introgression through hybridisation. We can directly test these alternative scenarios using dated trees inferred from our alignments. In order to provide comparable trees, we used the divergence date between the silvaniform and melpomene-cydno clades derived from a recent species tree for the Heliconinii [14] to calibrate the *dennis* and *ray* region phylogenies. These trees support introgression and rule out ancestral polymorphism because the dates of coalescence of the *H*. *melpomene* and *H*. *elevatus dennis* and *ray* alleles are significantly more recent than the divergence of these two species. These species last shared a common ancestor at around 3.96 Ma (95% highest posterior density [HPD] interval 3.18–4.81 Ma) [14]. In contrast, the *dennis* allele shared between *H*. *elevatus* and *H*. *melpomene/timareta* diverged around 1.95 Ma (2.79–1.25 Ma HPD). The divergence of the *ray* allele is even more recent and shared a common ancestor between *H*. *elevatus* and *H*. *melpomene/timareta* around 0.66 Ma (0.93–0.43 Ma HPD). The recent origin of these alleles is also supported by low levels of genetic diversity within these clades, with the average pairwise sequence divergence among the *dennis* alleles only 1.5%, including those from *H*. *melpomene*, *H*. *timareta*, and the more distantly related *H*. *elevatus* (Table 1, top). This is less than that found among the same individuals in flanking sequence (2.4%) and comparable to that among the red-forewing-banded “postman” group for the same locus (1.6%), which includes only more closely related *melpomene* and *timareta* individuals. The *ray* alleles also show only 1.1% average pairwise sequence divergence at the *ray* locus, similarly less than in the postman group at the same locus. Although sequence diversity is likely to be reduced in these regions because of functional constraint, it seems likely that such constraint is similar across different clades in the phylogeny, so the relatively low levels of diversity within the *ray* and *dennis* clade support their recent origin.

**Table 1:** Average pairwise sequence divergence between alleles within the two colour pattern-determining regions (%).

**DENNIS**	Outgroup	Silvaniforms	Dennis	Postman
Outgroup	**14.5**	17.6	17.5	16.9
Silvaniforms		**3.8**	4.1	5.3
Dennis			**1.5**	5.3
Postman				**1.6**
**RAY**	Outgroup	Silvaniforms	Ray	Postman
Outgroup	**13.6**	16.7	17.5	17.5
Silvaniforms		**3.6**	7.0	7.3
Ray			**1.1**	2.7
Postman				**1.2**
**FLANK—D**	Outgroup	Silvaniforms	Dennis	Postman
Outgroup	**16.0**	16.5	17.0	16.8
Silvaniforms		**3.2**	3.8	3.6
Dennis			**2.4**	2.3
Postman				**1.1**

Individuals were grouped by their phenotype rather than taxonomy, and average percentage pairwise divergence was calculated within either the *dennis* (top) or *ray* (middle) region alignments. All silvaniforms are grouped together except for *H*. *elevatus*, which is grouped with the dennis/ray *melpomene* and *timareta* morphs. As a control, a flanking region distal to the *dennis* allele relative to *optix* (see Fig 3) is also shown (bottom). The *dennis* and *postman* alleles are equally different to the silvaniforms for sequences outside the putative *dennis* region, as would be expected from the species tree. See S2 Table for divergences of ray individuals at the *dennis* region and dennis individuals at the *ray* region.

**Fig 5:**
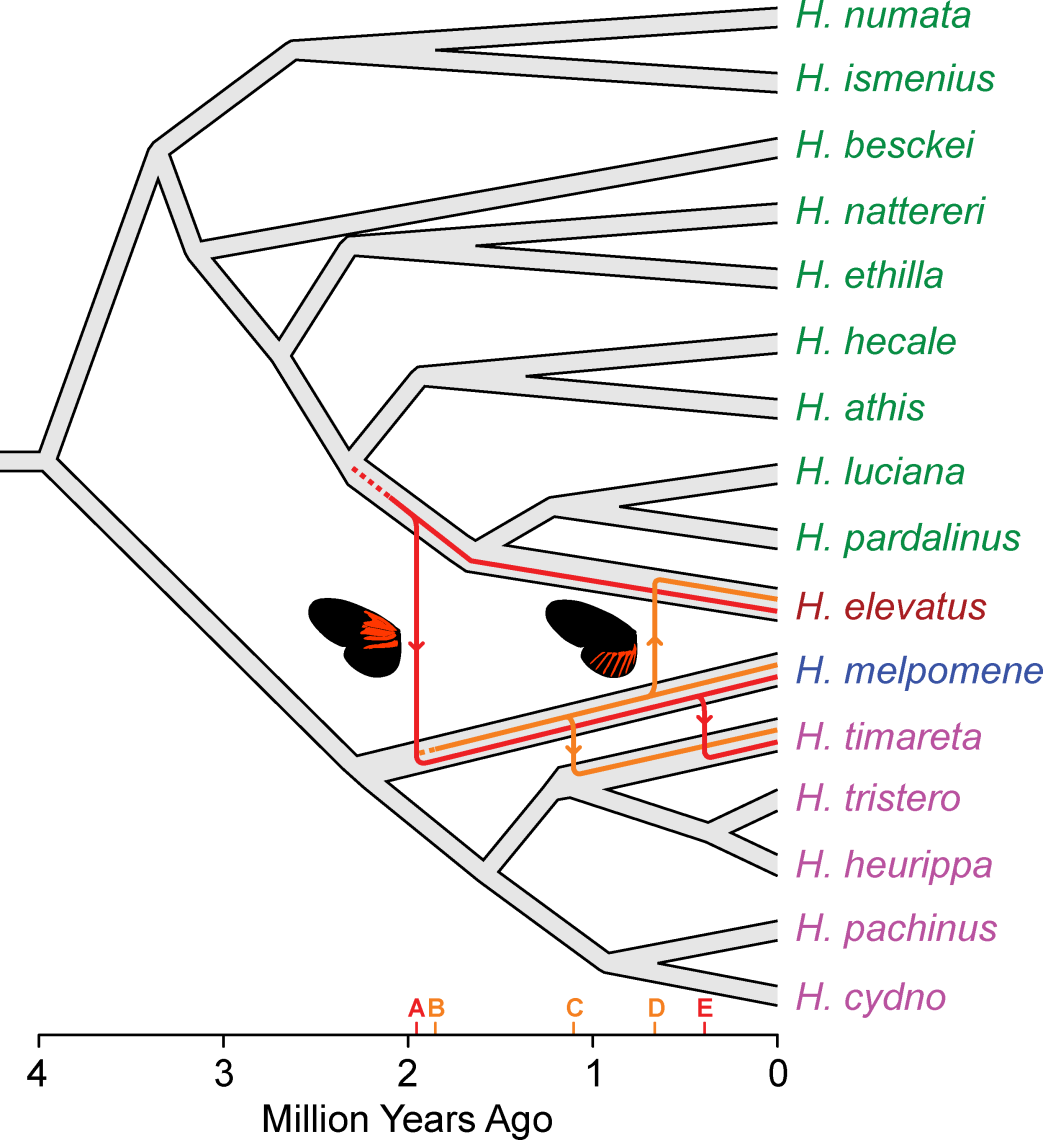
Hypothesis for the origins and introgression of the *dennis* and *ray* regions inferred from dated trees. Key events indicated on the x-axis are (A) introgression of *dennis* from *H*. *elevatus* into *H*. *melpomene*, inferred from the coalescence time of alleles sampled from these two species; (B) the origin of *ray* alleles within *H*. *melpomene*, inferred from the coalescence of *dennis* and non-*dennis* alleles within *H*. *melpomene*; (C) introgression of *ray* into *H*. *timareta*; (D) introgression of ray into *H*. *elevatus*, and (E) introgression of *dennis* into *H*. *timareta*. Dating of phylogenies was carried out using BEAST, and full dated trees with support limits are provided in S3 Fig. The species tree is derived from [14].

Our dated trees can also be used to infer the relative timing of introgression events. Here the data indicate that the *ray* allele originated within *H*. *melpomene* at around the same time as *dennis* introgressed into the *H*. *melpomene* clade from an ancestor of *H*. *elevatus*, sometime around 1.85 Ma (2.53–1.25 Ma HPD; Fig 5 and S3 Fig). This suggests that the characteristic mimetic dennis-ray phenotype first came together within *H*. *melpomene* at that time. In contrast, *H*. *elevatus* did not acquire the *ray* allele until about a million years later and perhaps persisted during this time as part of the Guiana Shield dennis-only mimicry ring. The *dennis* and *ray* alleles of *H*. *timareta* are each nested within those of *H*. *melpomene* and are more recent than the divergence of these species, implying introgression from *H*. *melpomene* into *H*. *timareta* and consistent with previous analyses [33]. Nonetheless, *dennis* and *ray* events also differ in timing, as most of the *H*. *timareta ray* alleles diverge from their *H*. *melpomene* relatives around 1 Ma, but the *dennis* alleles diverged only around 0.45 Ma (Fig 5 and S3 Fig). *H*. *timareta ray* alleles are polyphyletic with respect to *H*. *melpomene*, also supporting multiple introgression events and recombination between the regions. Further sampling will be needed to resolve more clearly the timing and number of introgression events between these species.

In addition to recombination between lineages, there is also shuffling of alternate alleles at these regulatory modules within species. Across most of their range, *H*. *melpomene* and *H*. *timareta* have either *postman* or *dennis-ray* haplotypes across the entire studied region. However, the race *H*. *m*. *meriana* has *dennis* alleles but shows recombination across the adjacent *ray* locus that removes the ray phenotype. Similarly, a single recombination event in *H*. *t*. *timareta* f. *contigua* produced a phenotype with ray but not dennis (Fig 2D; Fig 4). *H*. *elevatus*, like *H*. *melpomene*, also has a dennis-only race found in the Guiana Shield, which likely represents another case of enhancer shuffling within species, although we have not sampled this species here (Fig 4). Hence, although alleles within these regulatory modules are now highly divergent and presumably arose through accumulation of a number of mutations of small effect, novel phenotypes could have arisen rapidly through recombination between modules both within and between species.

We have demonstrated several aspects of the genetic architecture of wing pattern that have contributed to evolutionary innovation in the *Heliconius* radiation. First, distinct genetic elements are associated with different patches of red on the butterfly wing. This supports the hypothesis of regulatory modularity, which should facilitate evolutionary innovation. Second, the origin of the dennis-ray phenotype in *H*. *melpomene* involved a combination of evolutionary tinkering of existing patterns and introgression between species. Finally, we show that diversity within three lineages (*H*. *elevatus*, *H*. *melpomene*, and *H*. *timareta*) has been generated by shuffling of these distinct regulatory modules among populations and species. Within all three lineages, some populations possess one or other of these elements, providing further flexibility in pattern evolution. Our data imply that recombination between lineages can generate novel phenotypic combinations and demonstrate how modularity in the *cis*-regulatory control of key genes can drive the rapid evolution of novel morphologies. Although the evolution of novel regulatory modules may involve many mutational steps [5], these can subsequently be exchanged between lineages and shuffled into new combinations enabling rapid adaptive evolution. Recent studies showing that adaptation can proceed via gene flow of preadapted genetic modules between nearby populations or species suggest that similar mechanisms may be important in other radiations. In sticklebacks, adaptation to freshwater involves movement of alleles through the marine landscape [34]. Mosquitoes, Darwin’s finches, and even humans also show evidence for introgression of alleles between species that facilitate adaptation [35–37]. The extent to which recombination between regulatory alleles can contribute to morphological novelty in these other groups of organisms remains to be seen.

## Materials and Methods

### Genome Sequencing and Analysis

#### Specimen collection

Wings of field caught *Heliconius* butterflies were removed and stored in labelled envelopes and bodies preserved at −20°C in 20% DMSO, 0.25 M EDTA, salt saturated solution. For sample locations and phenotypes of *Heliconius* species collected, see S3 Table. DNA was isolated from one third of a thorax, yielding ~3 μg. Tissue was homogenised in buffer ATL using the TissueLyser (Qiagen) set for 4 min at 25 Hz, and DNA purified using the DNeasy Blood and Tissue Kit (Qiagen). RNA was removed with RNase A.

#### Sequencing and alignment

Whole genome shotgun sequences were generated for 93 samples using the Illumina HiSeq2000 platform, generating 100 bp, paired-end sequences (The Genepool, United Kingdom; Baylor College of Medicine Human Genome Sequencing Center, United States; FAS Center for Systems Biology, Harvard, US). The sequence for the HmD colour pattern region, obtained previously from 45 additional samples using targeted sequence capture (SureSelect, Agilent Technologies), was also analysed [28].

Raw reads from resequenced genomes were aligned to the *H*. *melpomene melpomene* reference (v1.1) using Stampy (v1.0.17) [38], with substitution rates of 0.03, 0.04, and 0.05 for *H*. *melpomene*, *H*. *timareta/heurippa*/*cydno*, and silvaniform samples, respectively. Duplicate reads were removed with Picard (v1.67, http://picard.sourceforge.net). The HE670865 HmBD colour pattern scaffold was extracted from genome BAM files using the Samtools *view* function, and each sample sorted and indexed. To minimise alignment errors around insertions and deletions, BAM files were combined and analysed using the Genome Analysis Tool Kit (GATK V 1.6–11) function RealignerTargetCreator to produce a single *intervals* file. The GATK function IndelRealigner was then used to correct read mapping inconsistencies around in/del regions, and then UnifiedGenotyper created a vcf file with the parameters -out_mode EMIT_ALL_CONFIDENT_SITES -baq CALCULATE_AS_NECESSARY -hets 0.01.

#### Genotype-phenotype association testing

Genotypes of 96 individuals were extracted from vcf files using a custom perl script with a minimum mapping quality of 30, genotype quality of 30, minimum read depth 4, and maximum depth of 300. Sites failing these thresholds were scored as a missing genotype, N. Chi-squared tests for association between genotype and phenotype were performed with R program (R Development Core Team, 2011) package GenABEL, using the *ccfast* function. Plots (Fig 2B) show *p*-values with one degree of freedom, with Bonforroni correction for 219,501 informative sites (−log_10_ ~6.64).

#### Fixed nucleotide differences between samples

Fixed nucleotide differences between two sample groups were identified using a custom python script (SM), and their distribution was plotted against scaffold HE670865 using R (R Development Core Team, 2011). Plots display the number of fixed sites within a 5 kb sliding window, moving at 1 kb intervals.

#### Fosmid and BAC sequencing

A *H*. *m*. *aglaope* fosmid library was screened using a DNA probe amplified using PCR primers BD_probe_F 5ʹ-AAAGTAGTCGGGTGCGCTTA-3ʹ BD_probe_R 5ʹ-CTGACTCGACATCCCTGTCA-3ʹ. Clone 1048-3N15 was purified, sheared with sonication (Bioruptor, Diagenode), subcloned into pGEM-t-Easy vector (Promega), and shotgun sequenced using an ABI3730 DNA Analyzer (Accession KU200223). The 36 Kb clone was assembled into five contigs from 302 reads, and the ends were ordered based on the orientation of the vectors used to create the fosmid library. Internal contigs were ordered based on evidence from a *H*. *m*. *agalope* (aglaope.1) de novo genome assembly using ABySS with a k-mer of 41. A second screened clone, 1048-143N20, was partially sequenced by PCR amplification of the *dennis* region (S4 Table), su-cloned into pGEM-t-Easy vector, and Sanger sequenced using standard T7F and SP6 primers. The BAC sequence for AEHM-19L14 was sequenced previously and downloaded from NCBI GenBank [39].

### Recombination Breakpoint Mapping

The paired-end sequencing reads for the 96 individuals used above plus an additional 43 individuals mainly representing outgroup species (S3 Table) were imported into CLC Genomics Workbench v5.5 and de novo assembled into contigs using default parameters (mismatch: 2; insertion: 3; deletion: 3; length fraction: 0.5; similarity fraction: 0.8). The resulting contigs were imported into Geneious v6.1 as FASTA files. These were used to construct BLAST databases in Geneious for each individual. Two further individuals were sampled in the form of the fully assembled reference sequences for *dennis* and *ray* regions, which were acquired from the *H*. *melpomene* reference genome v.1.1[16] and the fosmid 1048-3N15 from *H*. *m*. *aglaope*, which provided a reference sequence for the *dennis* haplotype. In Geneious, these sequences were then used as references to conduct BLASTn searches against the contig sequence databases for each of the 139 de novo assemblies. The BLAST results were then mapped back onto the reference sequence. Using the reference as a template, matching contigs were concatenated into a single FASTA file for each individual, with Ns filling regions between contigs spaced according to the reference (or closest relative). Unresolvable genomic repeats were detected as regions in which more than two haplotypes matched by BLAST and were replaced with Ns. Heterozygous haplotypes, in which just two contigs aligned, were assigned to two distinct FASTA versions of the region. Phasing of adjacent haplotypes was arbitrarily assigned, except for individuals heterozygous for phenotype, such as hybrid *H*. *melpomene*, in which heterozygous contigs could be clearly assigned to either dennis-ray or banded clades. In total, the final alignment included sequence derived from 142 individuals, including 139 de novo assembled genomes, two fosmid clones, a reference BAC sequence, and the reference genome.

#### Multiple alignment and tree generation

The compiled sequences of contigs for the *dennis* and *ray* regions for each individual were aligned using MAFFT in Geneious v6.1. (E-INS-i algorithm; Gap open penalty: 1.53). ML trees were generated using the PHYML Geneious plug-in (GTR substitution model; SPR topology search; 1,000 bootstraps).

#### Average pairwise divergence analysis

The multiple alignments generated for the *dennis* and *ray* region were analysed using MEGA 6.0 (http://www.megasoftware.net/). The average pairwise divergence was calculated for the following groups of taxa—outgroups: *H*. *aoede*, *H*. *hecuba*, *H*. *hierax*, *H*. *xanthocles*, *H*. *doris*, and *H*. *wallacei*; silvaniform group: *H*. *numata bicoloratus*, *H*. *n*. *silvana*, *H*. *n*. *tarapotensis*, *H*. *n*. *elegans*, *H*. *ethilla*, *H*. *pardalinus* ssp. *nov*., *H*. *p*. *sergestus*, *H*. *ismenius*, and *H*. *hecale*; dennis group: *H*. *e*. *pseudocupidineus*, *H*. *e*. *bari*, *H*. *m*. *thelxiopeia*, *H*. *m*. *meriana*, *H*. *m*. *ecuadorensis*, *H*. *m*. *malleti*, *H*. *m*. *aglaope*, and *H*. *t*. *florencia*; ray group: *H*. *e*. *pseudocupidineus*, *H*. *e*. *bari*, *H*. *m*. *thelxiopeia*, *H*. *m*. *ecuadorensis*, *H*. *m*. *malleti*, *H*. *m*. *aglaope*, *H*. *t*. *florencia*, and *H*. *t*. *timareta* f. *contigua*; and postman group: *H*. *m*. *melpomene*, *H*. *m*. *rosina*, *H*. *m*. *amaryllis*, *H*. *m*. *amandus*, *H*. *m*. *vulcanus*, *H*. *m*. *cythera*, *H*. *m*. *bellula*, and *H*. *t*. *thelxinoe*

#### Bayesian tree dating analysis

Following the methodology used in Kozak et al. [14], the multiple alignments generated for *dennis* and *ray* regions were prepared using the BEAST input file formatting tool, BEUti v1.8.2 [40]. The trees were calibrated by providing a nodal age prior to the silvaniform and melpomene-cydno clade split, with a mean of 3.96 Ma and an interval of 3.18–4.81 Ma, as predicted for that node by Kozak et al. [14]. The tree prior used the “speciation: birth-death process” [41], and the MCMC chain was run for 10,000,000 states, with a 20% burn-in. The chain was run using BEAST v1.8.2 and checked for MCMC convergence using the diagnostic tool Tracer v1.5 [42]. The output tree data were then compiled using TreeAnnotator v1.8.2, and the 95% HPD intervals were visualised using TreeFig v1.4.2 [43]. Sequence data files have been submitted to the European Nucleotide Archive with project number ERP009041.

All software are available at http://tree.bio.ed.ac.uk/software/.

## Supporting Information

S1 Fig Fixed nucleotide sites associated with the ray and dennis phenotypes in scaffold HE670865. Pairwise sequence comparison using *H*. *melpomene* and/or *H*. *timareta* specimens grouped by phenotype reveals narrow regions associated with specific wing patterning phenotypes. Plots show the number of fixed nucleotide sites within a 5 kb sliding window (at 1 kb intervals) on scaffold HE670865, between two phenotypic groups (S1 Table). Protein coding exons spanning the scaffold, including *optix*, are shaded black. (A) Sequence comparison of ray and non-ray groups identified a minimal region of fixed nucleotide differences between 354,278 and 372,171 bp. (B) A comparison between dennis and non-dennis expressing phenotypes identified a minimal region of fixed nucleotide differences between 325,007 and 329,296 bp. (C) Sites associated with the ray and dennis phenotype are non-overlapping. See S1 Table, below, for samples used in pairwise comparisons. See Dryad depository for plot data [32].
(TIF)

S2 Fig Phylogenies for multiple alignments of *dennis* and *ray* regions. Labelled trees from Fig 2B, showing dennis morphs including *H*. *elevatus* (tree A) clustering with the silvaniform clade and ray morphs including *H*. *elevatus* (tree B) clustering with the *H*. *melpomene* and *H*. *timareta* clade. Individuals that have dennis-only pattern (D) group with dennis morphs (tree A), but not with ray morphs (tree B). The reverse is true of ray-only morphs (R). ML trees rooted to *H*. *aoede* outgroup, bootstraps = 1,000 iterations. See Dryad depository for ML treefiles [32].
(TIF)

S3 Fig Dated phylogenies for multiple alignments of *dennis* and *ray* regions with dated species tree for comparison. The nodes for trees generated from the dennis and ray alignments were dated using BEAST MCMC software, and the 95% HPD interval is depicted with horizontal bars. The clades are coloured according to S2 Fig, with the addition of the dennis-ray morph timareta, *H*. *t*. *florencia*, in light brown. Also shown is the equivalent portion of the species tree from Kozak et al. [14], with all dates given in Ma. Major events shown on Fig 5 in the main text are also marked here for comparison, with (A) showing introgression of *dennis* from *H*. *elevatus* into *H*. *melpomene*, inferred from the coalescence time of alleles sampled from these two species, and the recency of this compared to species tree divergence of silvaniform and melpomene clades at 3.96 Ma; (B) marking the origin of *ray* alleles within *H*. *melpomene*, inferred from the coalescence of *dennis* and non-*dennis* alleles within *H*. *melpomene*; (C) marking introgression of *ray* into *H*. *timareta*; (D) introgression of ray into *H*. *elevatus*, and (E) marking introgression of *dennis* into *H*. *timareta*. See Dryad depository for dated treefiles with node values [32].
(TIF)

S1 Table Specimens used to conduct pairwise sequence comparisons for identification of sites associated with the ray and dennis phenotypes (S1 Fig and Fig 2C). (XLSX)

S2 Table Additional nucleotide similarity matrices. Identical nucleotide similarity comparison was conducted between alleles for the *dennis* and *ray* regions as shown in Table 1, but with ray-morph individuals grouped together for the *dennis* region analysis (top) and dennis-morph individuals grouped for the *ray* region analysis (bottom).
(XLSX)

S3 Table List of samples used for sequencing. (XLSX)

S4 Table Seven pairs of primer sequences used to amplify sequential fragments from the *dennis* region of fosmid clone 143N20. (XLSX)

## Abbreviations

BLAST: Basic Local Alignment Search Tool
HPD: highest posterior density
ML: maximum likelihood
SNP: single nucleotide polymorphism
