# Supplementary figures and images for: Evolutionary Novelty in a Butterfly Wing Pattern through Enhancer Shuffling
Source: PLoS Biol. 2016 Jan 15;14(1):e1002353. doi: 10.1371/journal.pbio.1002353 (PMC4714872; doi:10.1371/journal.pbio.1002353)

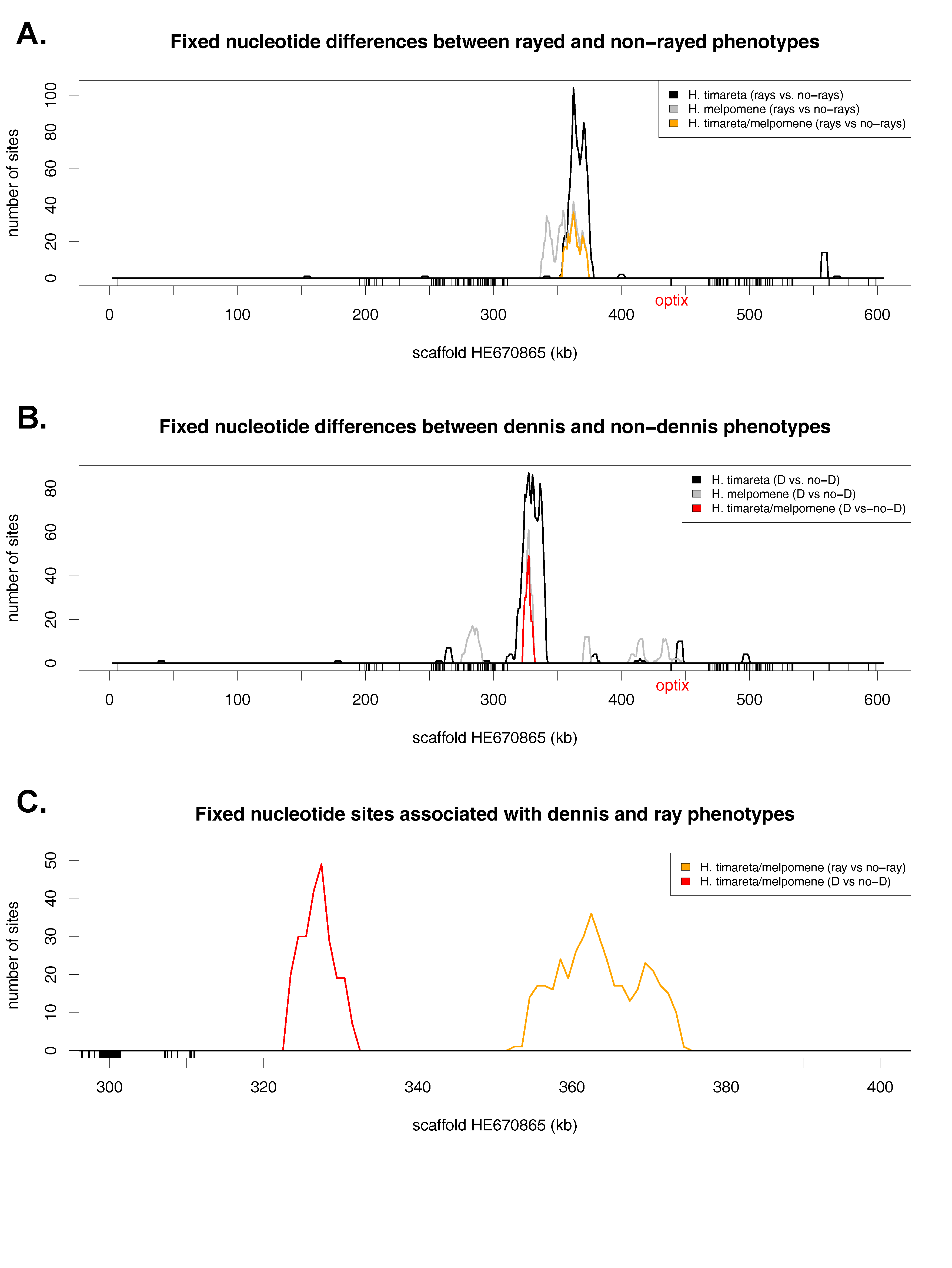

Supplement: S1 Fig — Pairwise sequence comparison using H. melpomene and/or H. timareta specimens grouped by phenotype reveals narrow regions associated with specific wing patterning phenotypes. Plots show the number of fixed nucleotide sites within a 5 kb sliding window (at 1 kb intervals) on scaffold HE670865, between two phenotypic groups (S1 Table). Protein coding exons spanning the scaffold, including optix, are shaded black. (A) Sequence comparison of ray and non-ray groups identified a minimal region of fixed nucleotide differences between 354,278 and 372,171 bp. (B) A comparison between dennis and non-dennis expressing phenotypes identified a minimal region of fixed nucleotide differences between 325,007 and 329,296 bp. (C) Sites associated with the ray and dennis phenotype are non-overlapping. See S1 Table, below, for samples used in pairwise comparisons. See Dryad depository for plot data [32]. (TIF) [file pbio.1002353.s001.tif]

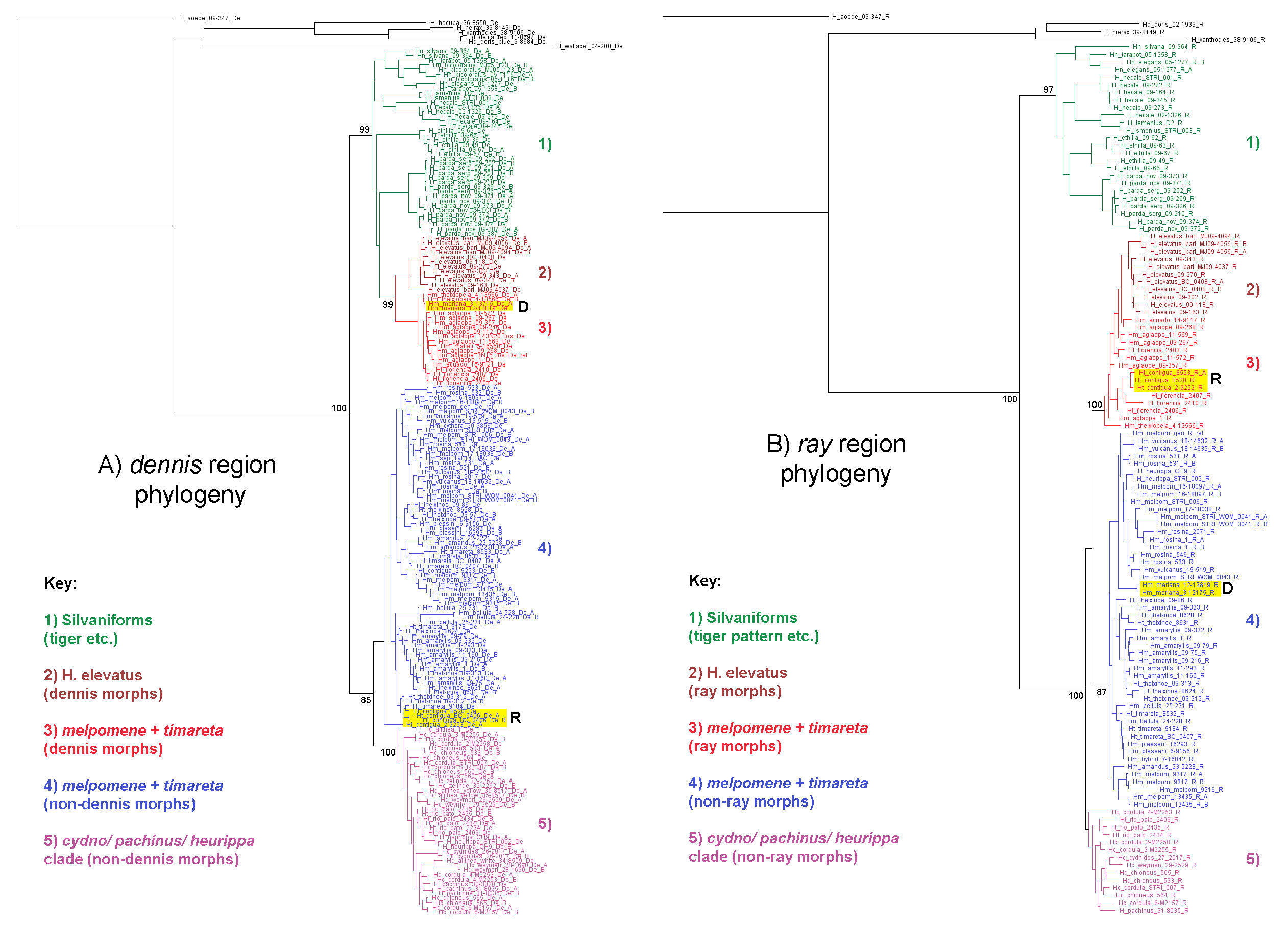

Supplement: S2 Fig — Labelled trees from Fig 2B, showing dennis morphs including H. elevatus (tree A) clustering with the silvaniform clade and ray morphs including H. elevatus (tree B) clustering with the H. melpomene and H. timareta clade. Individuals that have dennis-only pattern (D) group with dennis morphs (tree A), but not with ray morphs (tree B). The reverse is true of ray-only morphs (R). ML trees rooted to H. aoede outgroup, bootstraps = 1,000 iterations. See Dryad depository for ML treefiles [32]. (TIF) [file pbio.1002353.s002.tif]

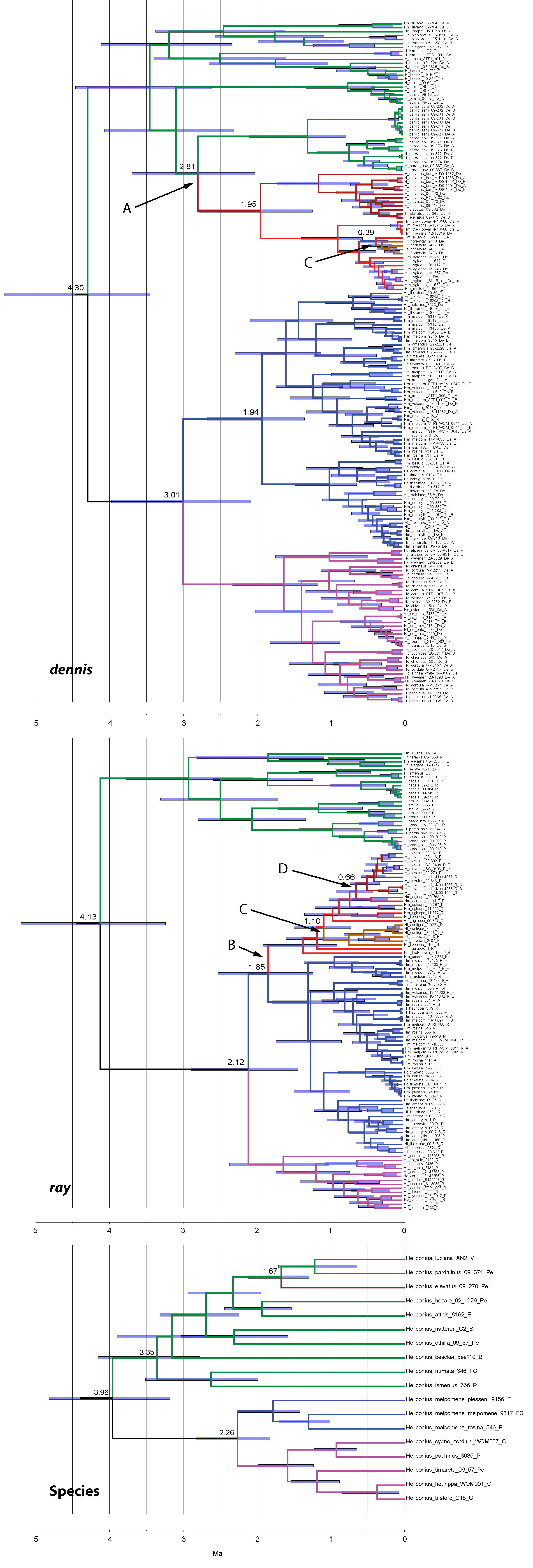

Supplement: S3 Fig — The nodes for trees generated from the dennis and ray alignments were dated using BEAST MCMC software, and the 95% HPD interval is depicted with horizontal bars. The clades are coloured according to S2 Fig, with the addition of the dennis-ray morph timareta, H. t. florencia, in light brown. Also shown is the equivalent portion of the species tree from Kozak et al. [14], with all dates given in Ma. Major events shown on Fig 5 in the main text are also marked here for comparison, with (A) showing introgression of dennis from H. elevatus into H. melpomene, inferred from the coalescence time of alleles sampled from these two species, and the recency of this compared to species tree divergence of silvaniform and melpomene clades at 3.96 Ma; (B) marking the origin of ray alleles within H. melpomene, inferred from the coalescence of dennis and non-dennis alleles within H. melpomene; (C) marking introgression of ray into H. timareta; (D) introgression of ray into H. elevatus, and (E) marking introgression of dennis into H. timareta. See Dryad depository for dated treefiles with node values [32]. (TIF) [file pbio.1002353.s003.tif]
